# Truview EVO2 and Standard Macintosh Laryngoscope for Tracheal Intubation During Cardiopulmonary Resuscitation: A Comparative Randomized Crossover Study

**DOI:** 10.1097/MD.0000000000000078

**Published:** 2014-09-19

**Authors:** Ewelina Gaszynska, Tomasz Gaszynski

**Affiliations:** Department of Hygiene and Health Promotion (EG); and Department of Emergency Medicine and Disaster Medicine, Medical University of Lodz, Lodz, Poland (TG).

## Abstract

The aim of this study was to compare the performance of the Truview EVO2 laryngoscope in manikin-simulated cardiopulmonary resuscitation (CPR) and no-CPR scenarios with standard intubation technique.

Participants performed 4 scenarios in random order: endotracheal intubation (ETI) using Macintosh laryngoscope (MCL), Truview EVO2 laryngoscope in no-CPR patient scenario, and intubation during uninterrupted chest compressions using both laryngoscopes. The participants were directed to make 3 attempts in each scenario. Primary outcomes were time to tracheal intubation (TTI) and intubation success, whereas secondary outcomes were cumulative success ratio and the number of esophageal intubation (EI). TTI and success ratios were reported per attempt.

Thirty paramedics completed the study. Median TTI with Truview EVO2 with CPR was 36 (interquartile range [IQR] 29.00–52.00), 22.5 (IQR 18.33–35.00), and 18 (IQR 11.00–23.00) seconds; MCL with CPR was 23 (IQR 18.92–36.90), 16.8 (IQR 14.00–22.31), and 14.5 (IQR 11.12–16.36) seconds; Truview EVO2 without CPR was 28.6 (IQR 24.02–38.34), 21.7 (IQR 17.00–25.00), and 13 (IQR 11.90–17.79) seconds; MCL without CPR was 17 (IQR 13.23–22.29), 13 (IQR 12.09–15.26), and 12.4 (IQR 10.08–19.84) seconds for first, second, and third attempts, respectively. The *P* values for differences in TTI between Truview EVO2 and MCL were *P* < 0.0001, *P* = 0.0540, and *P* = 0.7550 in CPR scenario and *P* = 0.0080, *P* = 0.1570, and *P* = 0.7652 in no-CPR scenario for first, second, and third attempts, respectively. The success ratios for each of the scenarios were as follows: in CPR scenario it was 0.73 versus 0.53 (*P* = 0.0558), 0.83 versus 0.76 (*P* = 0.2633), and 1 versus 0.8 (*P* = 0.0058); in no-CPR scenario it was 0.63 versus 0.73 (*P* = 0.2068), 0.86 versus 0.86, and 0.97 versus 1 (*P* = 0.1637) for Truview EVO2 vs MCL in first, second, and third attempts, respectively.

The cumulative success ratio related to the time of ETI was better for MCL compared with Truview EVO2 laryngoscope in both scenarios (*P* = 0.0029 and *P* = 0.0004 in no-CPR and CPR scenarios). The number of EI with MCL was 30% versus 13.3% (*P* = 0.0113), and for Truview EVO2 it was 20.45% versus 15.56% in CPR and no-CPR scenarios, respectively.

The application of Truview EVO2 during uninterrupted chest compressions increased TTI but increased the success ratio of ETI and decreased number of EIs.

## INTRODUCTION

Cardiopulmonary resuscitation (CPR) is recommended to be continued during airway attempts, and the current standard is endotracheal intubation (ETI). It is strongly advised by the European Resuscitation Council not to interrupt chest compressions during CPR for prolonged airway management attempts in order to maximize coronary and cerebral perfusion pressure.^1^ Only brief pauses in chest compressions are allowed to pass the tube through the vocal folds. Skilled providers should even try to perform intubation with no interruptions to chest compressions at all.^1^ Previous reports showed that with chest compressions the time required for successful ETI was longer.^2^ Several studies on new devices for ETI testing for efficacy during CPR are accessible.^3^

None of these studies have examined the Truview EVO2 for CPR. The standard method of definitive airway management is ETI with a standard Macintosh laryngoscope (MCL). Chest compressions may deteriorate the laryngeal view. Therefore, CPR makes intubation more demanding. Videolaryngoscopes may assist in ETI during CPR,^4^ although this has not been adequately studied in clinical settings. There have also been very few studies in the emergency medical service (EMS) population. For paramedics who do not intubate regularly, videolaryngoscopes are a reasonable option for emergency airway management as they have been shown to allow for a significantly higher success ratio.^4^ With highly experienced providers like anesthesiologists, the advantage of videolaryngoscopes over standard laryngoscope is not observed.^5^

**FIGURE 1:**
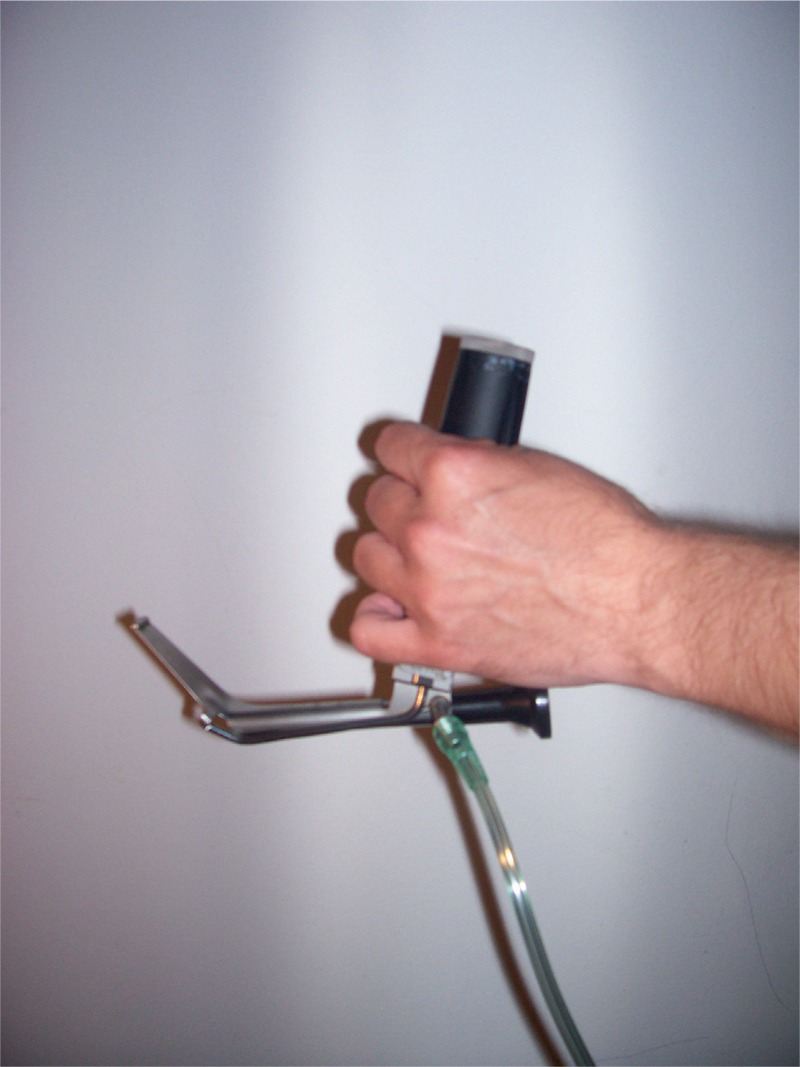
Truview EVO2 laryngoscope with catheter for constant oxygen delivery to the tip of blade.

Truview EVO2 (Truphatek, Netanya, Israel) is a novel optical laryngoscope with modified blade. Laryngeal view is obtained by an optical view tube incorporated into the blade (Figure 1). Similar to videolaryngoscopes, it makes it possible to visualize the larynx from a certain distance (about 20–30 cm). This allows to avoid close approach of the patient’s face, which may be helpful during emergency action such as resuscitation. The operator does not have to get close to the patient’s face and the Truview EVO2 provides wider view of entrance to the larynx, which may influence success ratio of intubation attempts. The Truview EVO2 laryngoscope’s blade has a channel for constant oxygen delivery to the tip of the blade, which prevents the lens from fogging as well as removes any secretions. The Truview EVO2 blade has a shape more similar to the Miller blade, but the laryngoscope itself is operated more like videolaryngoscope; it should be inserted into the mouth of the patient in the middle line while elevating the tongue (instead of pushing it to the left side). The objective of this study was to compare TruView with standard ETI in simulated patients who required CPR and did not require CPR.

We hypothesize that Truview EVO2 laryngoscope reduces time to tracheal intubation (TTI), increases intubation success rate, and reduces the number of esophageal intubations (EIs) as compared with standard MCL. The aim of the study was to evaluate if it is observed only during intubation attempts with uninterrupted chest compressions. Therefore, we compared its performance also in the no-CPR scenario. We performed a manikin crossover study comparing Truview EVO2 and standard MCLs in CPR and no-CPR scenarios.

## METHODS

### Setting and Sample

In EMS system in Poland, paramedics serve in basic ambulances. In Poland, there are 3 types of ambulances: basic life support (or rescue) ambulance with 2 paramedics, specialist or advanced life support ambulance with medical doctor and paramedics; and transport ambulance with driver only. The paramedics are allowed to perform ETI only for CPR. Thirty paramedics voluntarily took part in the study. They all had previous experience in ETI as they work in ambulances and perform ETI only during cardiac arrest. All participants were informed about the purpose of the study, signed a written consent, and eventually received 30 minutes of training with the Truview EVO2 laryngoscope provided by investigators on the same manikin that was used during the study. The study protocol was approved by the ethics committee of the Medical University of Lodz (Protocol number, RNN/607/10/KB; chairperson, Prof P. Polakowski; on October 12, 2010).

### Data Collection

In our study, we employed 2 different scenarios: ETI with a standard MCL or with Truview EVO2 laryngoscope, performed on an immobile manikin (no-CPR scenario) followed by ETI attempts with the same devices during uninterrupted chest compressions (CPR scenario). The manikin used was Ambu MegaCode Man (Ambu A/S, Ballerup, Denmark) with normal airway (no difficult airway simulation), lying in supine position on the ground.

There were 2 different scenarios for each equipment used: in the first scenario, the patient did not require CPR, whereas in the second scenario, CPR was required. Participants did each of 2-minute scenario type twice (a total of 4 scenarios each), in which they used either the Truview or MCL laryngoscope, the order of which was random. In each scenario, participants were directed to attempt intubation 3 times.

The investigator running the scenario told the participants when to make an attempt and recorded (using an IVT stopwatch; Conrad Electronic, Germany) the time elapsed from the moment the participant took hold of each device to effective manikin ventilation with a bag valve mask, confirmed by a volumeter on the manikin. A failed intubation attempt was defined as EI. The Ambu MegaCode manikin included sensors that indicated whether the tube was placed in esophagus. Size 3 blade of the MCL or the Truview EVO2 was used at random in each case. Every participant intubated the manikin 3 times in each scenario regardless of whether the attempts were successful or not. The intubation was performed in 1 session with randomly (closed envelope method) chosen order of device. Every session lasted 2 minutes. In all cases, a J-shaped stylet (similar to the Hilton et al^6^ model) and the same type of endotracheal tube (Sumi, Sulejowek, Poland) were used. The number of successful intubations and failures was recorded. For each attempt, all airway devices and the manikin’s airway were lubricated in accordance with the manufacturer’s instructions. The internal diameter of the tracheal tube was 7.5 mm. One participant continued chest compressions while the other performed ETI. The frequency of chest compressions was 100/min controlled by a timer. Compression depth of 4–5 cm was maintained according to the electronic measurement system of the Ambu MegaCode Man manikin.

### Data Analysis

Our primary endpoint of this study was the time (median and interquartile range) required for successful intubation for each laryngoscope—TTI. The secondary endpoints are cumulative success ratios related to time as they indicate which of the evaluated devices may influence the chest compression fraction and thus the surviving ratio and the number of EIs in each evaluated device. Cumulative success ratio was counted as percentage of successful intubations in time intervals in both scenarios.

Statistical analysis was performed with STATISTICA 10.0 software (StatSoft, Tulsa, OK). The χ^2^ test for independent pairs was used with the Yates correction if necessary (analysis of EI). The repeated measures of analysis of variance were used for multiple levels of clustering. Mann–Whitney *U* test was used for nonpaired categorical and continuous data analysis (for TTI in different CPR scenario). Post hoc testing was performed with the Fisher least significant difference test. Kaplan–Meier curves were drawn and a log-rank test was performed for group comparison (cumulative success ratio). *P* values <0.05 were considered statistically significant.

## RESULTS

**FIGURE 2:**
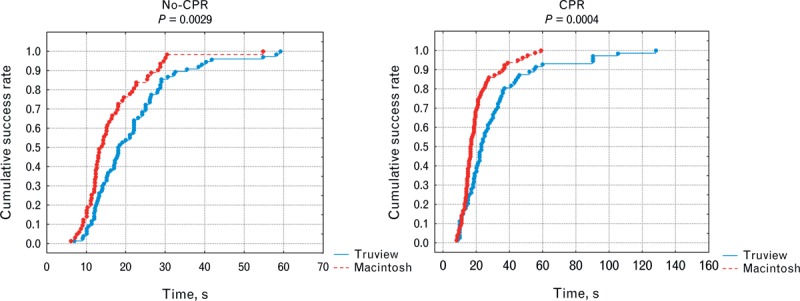
A comparison of cumulative success rate between Truview EVO2 and MCL in no-CPR and CPR scenarios. CPR = cardiopulmonary resuscitation, MCL = Macintosh laryngoscope.

Thirty paramedics (7 women and 23 men; age, 29 years, SD 6.9 [minimum 23, maximum 36]) with various professional experiences ranging from 2 to 15 years working in basic ambulance (mean experience, 6.95 years, SD 3.21) completed the study. Median TTI was significantly longer for Truview EVO2 compared with MCL for the first attempt only (Table 1). Chest compressions significantly prolonged (*P* < 0.05) TTI calculated for all attempts for both laryngoscopes (Table 1). TTI reduction for all 3 attempts in between CPR and no-CPR scenarios for the 2 examined laryngoscopes proved to be not significant (Table 1). However, when comparing times of attempts depending on scenario, there was a significant improvement in TTI for first, second, and third attempts for both laryngoscopes (Table 1)—next attempts did shorten TTI. The success ratio at first, second, and third attempts of ETI during uninterrupted chest compressions was higher for Truview EVO2 compared with MCL (Table 2). For the no-CPR scenario, success ratios at each of the 3 attempts was similar in both the laryngoscopes evaluated (Table 2, *P* = 0.29). The cumulative success rate related to the time of ETI was better for MCL compared with Truview EVO2 laryngoscope in both scenarios (Figure 2) (*P* = 0.0029 and *P* = 0.0004 in no-CPR and CPR scenarios, respectively). Overall evaluation of the influence of CPR on the number of EIs for both laryngoscopes revealed no significant difference: 16.67% versus 22.78% for no-CPR and CPR scenarios, respectively (*P* = 0.15). In MCL group, CPR significantly increased the incidence of EI (*P* = 0.0113) (Table 3). This difference was not significant for Truview EVO2 laryngoscope (*P* = 0.512) (Table 3). The application of Truview EVO2 laryngoscope significantly decreased the risk of esophagus intubation during CPR (odds ratio, 0.43; 95% confidence interval, 0.21–0.89).

**TABLE 1:**
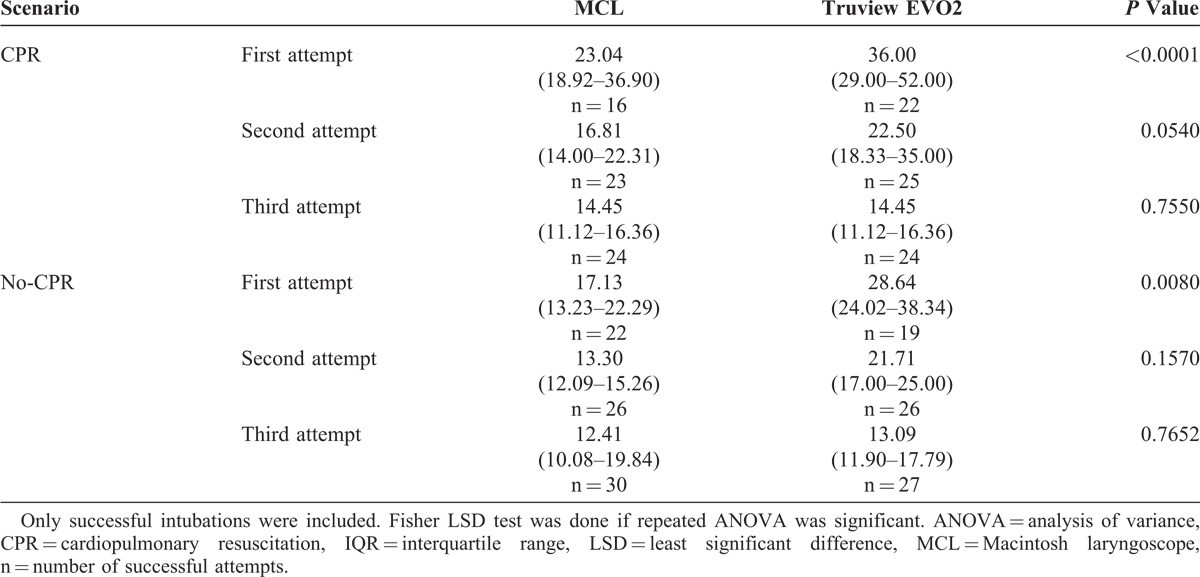
Median Time to Successful Intubation in Following 3 Attempts in CPR and No-CPR Scenarios: Median (IQR) [seconds]

**TABLE 2:**
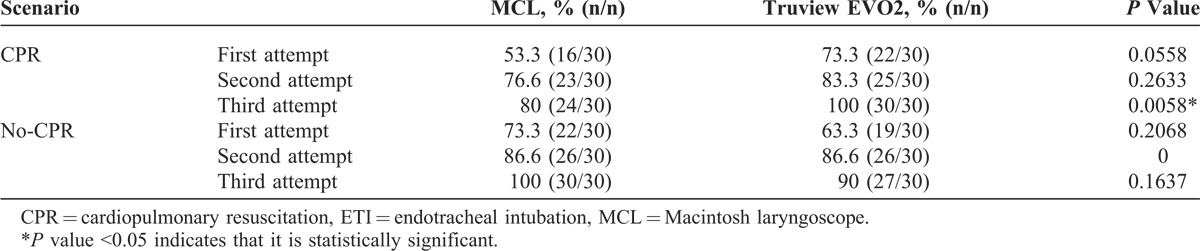
Success Ratio at First, Second, and Third Attempt of ETI During CPR and No-CPR Scenarios

**TABLE 3:**
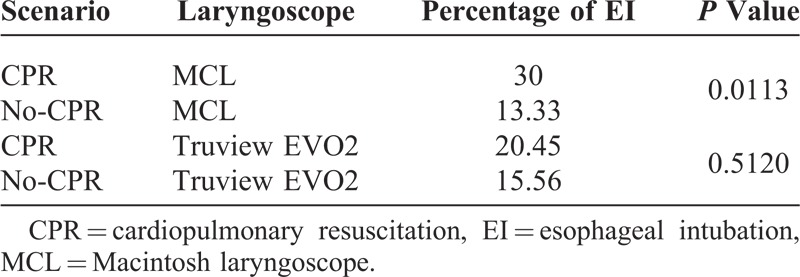
EIs (χ^2^ Yates Test)

## DISCUSSION

In this first report on the application of Truview EVO2 during uninterrupted chest compressions, we observed that the use of Truview EVO2 during CPR although increased TTI also increased the success ratio of ETI. Constant oxygen delivery by a channel incorporated in the Truview EVO2 laryngoscope blade may be advantageous in oxygenation of resuscitated patients.^7^ Results of some studies confirm that Truview EVO2 is useful in cases of difficult intubation.^8^ It enables better laryngeal visualization and reduces the TTI compared with the traditional MCL.^9^ In our study, the TTI was significantly longer for Truview EVO2 in the first attempt. This may be due to participants’ lack of experience with this new device. TTI in next attempts were similar between laryngoscopes, probably because the participants learned how to use them. The success ratio was higher for MCL in no-CPR scenario, but in CPR scenario Truview EVO2 performed better. This may suggest that Truview EVO2 may be recommended in clinical situations where intubation is potentially difficult because of chest compressions. The number of EI was smaller for Truview EVO2. Its operation as well as blade shape may be considered similar to GlideScope videolaryngoscope (Verathon, Amersham, UK). Still, remembering that Truview EVO2 is a different device, we may discuss the results of our study comparing them with studies performed with GlideScope. Xanthos et al^10^ tested GlideScope in conditions similar to those used by us and obtained different results: GlideScope was comparable to MCL during chest compressions in terms of TTI but had a higher success rate than MCL. In our study, the TTI with Truview EVO2 was longer compared with MCL, but we observed fewer EIs with the former. Kim et al^11^ evaluated whether chest compressions affected TTI, using 3 laryngoscopes operated by 20 paramedic students: MCL, GlideScope, and Airway Scope (Hoya, Tokyo, Japan). They found that TTI was not significantly affected by chest compressions, but cumulative success rates related to TTI were significantly higher for videolaryngoscopes. Our observations are similar for TTI, but the cumulative success rate was higher for MCL in our study. However, the incidence of EI was significantly lower with Truview EVO2. The incidence of EI in out-of-hospital setting may reach 30%, especially if performed by paramedics.^12,13^ In our study, we observed that uninterrupted chest compressions resulted in an increased incidence of EI with MCL but not with Truview EVO2 laryngoscope.

The studies comparing Truview laryngoscope with other devices reveal different conclusions. Malik et al,^14^ who compared Truview with MCL, GlideScope, and Pentax-AWS (Hoya, Tokyo, Japan) in simulated difficult scenarios on manikin models concluded that the Pentax-AWS laryngoscope demonstrated more advantages over the MCL than either the Truview EVO2 or the GlideScope laryngoscope, when used by experienced anesthetists in difficult tracheal intubation scenarios. Miceli et al^15^ in their manikin study found out that compared with the classical Macintosh blade, the Truview EVO2 blade allowed a better view of the larynx, but did not facilitate ETI in any of the difficult scenarios created with the adjustable manikin and in most scenarios in fact prolonged the intubation time. Both cited studies were performed with experienced anesthesiologists as participants and on no-CPR scenarios. In our opinion, experience in the use of a specific type of device influences results very much. A participant, who is using MCL for several years and has great experience in standard intubation, usually prefers MCL over new devices, and performs better ETI in difficult scenarios with MCL than using other new devices.^16^ In our study, participants had little professional experience with MCL or any other intubation device (paramedics intubate rarely comparing to experienced anesthesiologists), so our results reflect better in our opinion possible advantages and disadvantages of evaluated devices. The study by Tutuncu et al^17^ performed on patients revealed that the Truview EVO2 appears to be better than the Macintosh blade because of its continuous oxygen insufflation system that cleans the secretions, and its optical apparatus that significantly improves the view of the laryngeal entry. Timanaykar et al^18^ had the same observations and concluded that tracheal intubation using Truview blade provided consistently improved laryngeal view as compared with Macintosh blade without the need to align the oral, pharyngeal, and tracheal axes, with equal attempts for successful intubation, and similar changes in hemodynamics. However, the time taken for intubation was more with Truview, which is similar to our observation.

## STUDY LIMITATIONS

The presented study has several limitations. First of all, it is a manikin study. Because of technical and ethical issues, it may be difficult to perform a crossover study involving human subjects. Although manikin studies have their disadvantages (simulation lacked ecological validity to the normal EMS setting, difference in chest and other structures of manikin compliance compared with real tissue), they are still valuable in that they suggest which approach may be more beneficial to real patients. Second, the number of participants was limited due to the inability to recruit more at the time the study was conducted. A sample size calculation was not conducted and the sample was a convenience sample basing on literature.^11,19^ Kim et al^11^ performed a similar study on 22 participants with 1 attempt, and wrote, “based on the results of retrospective power calculation, our sample size has 82% power to detect differences among the laryngoscopes with a 0.05 significant level.”

Cho et al^19^ performed study on 24 participants, and wrote, “Sample size was calculated based on our pilot data of measurements of intubation time (16.4 ± 3.6 seconds). Twenty-four participants would be required to demonstrate a 20% difference in intubation time between intubation devices (b = 0.2; a = 0.05).” In our study, we had 30 participants involved.

Third, the participants had clinical experience only with MCL but not with Truview EVO2. They had trained intubation with Truview EVO2 only before the study on manikin model.

Many alternatives to direct laryngoscopy exist and, when evaluated in manikin studies, it is almost always demonstrated that they improve ETI success rate.^3,10–12^ However, in clinical practice, this may not matter if for example the time of intubation is few seconds shorter for studied device compared with MCL. In our study, we concentrated on another important problem of out-of-hospital intubations, which is unrecognized EI. In unrecognized EI, patients are ventilated through an endotracheal tube located in the esophagus and may not present deep hypoxia because they have either some spontaneous ventilation or a traumatic postintubation connection between esophagus and trachea. If we could decrease the number of EI by some device, in our opinion it may influence significantly the safety of patients. In our study, the number of EI was decreased by using Truview EVO2 by 10% compared with MCL.

## CONCLUSION

The Truview EVO2 laryngoscope was found to have a longer TTI than MCL in both CPR and no-CPR scenarios. The success rates were better for Truview EVO2 in the CPR scenarios and similar in no-CPR scenarios. Of the unsuccessful intubations, fewer EIs were noted for Truview EVO2 than MCL. These results may provide preliminary evidence for the potential use of Truview EVO2 as an alternative to MCL, but further clinical research is needed before it can be reliably recommended.

## ACKNOWLEDGMENT

The authors thank Ms Corrine Fiddick, MD (Canada) for language correction of the manuscript.
